# Factors for change in maternal and perinatal audit systems in Dar es Salaam hospitals, Tanzania

**DOI:** 10.1186/1471-2393-10-29

**Published:** 2010-06-03

**Authors:** Angelo S Nyamtema, David P Urassa, Andrea B Pembe, Felix Kisanga, Jos van Roosmalen

**Affiliations:** 1 Tanzanian Training Centre for International Health, Ifakara, Tanzania; 2 Department of Community Health, School of Public Health and Social Sciences, Muhimbili University of Health and Allied Sciences, Dar es Salaam, Tanzania; 3 Department of Obstetrics and Gynaecology, School of Medicine, Muhimbili University of Health and Allied Sciences, Dar es Salaam, Tanzania; 4 Department of Obstetrics, Leiden University Medical Centre, Leiden, The Netherlands; 5 Department of Metamedics, VU University Medical Centre Amsterdam, Amsterdam, The Netherlands

## Abstract

**Background:**

Effective maternal and perinatal audits are associated with improved quality of care and reduction of severe adverse outcome. Although audits at the level of care were formally introduced in Tanzania around 25 years ago, little information is available about their existence, performance, and practical barriers to their implementation. This study assessed the structure, process and impacts of maternal and perinatal death audit systems in clinical practice and presents a detailed account on how they could be improved.

**Methods:**

A cross sectional descriptive study was conducted in eight major hospitals in Dar es Salaam in January 2009. An in-depth interview guide was used for 29 health managers and members of the audit committees to investigate the existence, structure, process and outcome of such audits in clinical practice. A semi-structured questionnaire was used to interview 30 health care providers in the maternity wards to assess their awareness, attitude and practice towards audit systems. The 2007 institutional pregnancy outcome records were reviewed.

**Results:**

Overall hospital based maternal mortality ratio was 218/100,000 live births (range: 0 - 385) and perinatal mortality rate was 44/1000 births (range: 17 - 147). Maternal and perinatal audit systems existed only in 4 and 3 hospitals respectively, and key decision makers did not take part in audit committees. Sixty percent of care providers were not aware of even a single action which had ever been implemented in their hospitals because of audit recommendations. There were neither records of the key decision points, action plan, nor regular analysis of the audit reports in any of the facilities where such audit systems existed.

**Conclusions:**

Maternal and perinatal audit systems in these institutions are poorly established in structure and process; and are less effective to improve the quality of care. Fundamental changes are urgently needed for successful audit systems in these institutions.

## Background

The persistently high maternal mortality ratio (MMR) of 572/100,000 live births in Dar es Salaam [1] together with a hospital based MMR of 1602/100,000 live births in 2004 and a perinatal mortality rate (PNMR) of 123/1000 births from 1999 to 2003 at Muhimbili National Hospital have been noted with great concern [2,3]. From the Safe Motherhood Initiative perspectives, one of the simplest and cost effective strategic interventions to reduce maternal and perinatal deaths is to improve the quality of care in the existing health institutions [4]. This has been achieved through establishment of effective maternal and perinatal audit systems [5,6].

An effective audit system is a cycle that consists of identifying cases, collecting information, analyzing the results, formulating recommendations, implementing change and re-evaluating practice, and this cycle must be repeated regularly [7-9]. An audit panel looks into factors in the structure, process and outcome before giving its opinions. Structure is defined as the resources available for care, including the staff, equipment and facilities, and their organization. Process is the utilization of these resources in the provision of health care, and outcome refers to the result of the health care provision process [10]. In such reviews the audit panel determines the causes of death, areas of substandard care and any other preventable factors and recommends how to improve future management.

Spontaneous adoption of simple health practices is often very slow and the integration can take up to centuries after their effects are known if an internal change agent is not created [11,12]. Although maternal and perinatal audits at the level of care were formally introduced in Tanzania around 25 years ago [13], little is known about their existence, performance, and practical barriers to their implementation in Dar es Salaam. This study assessed the structure, process and impacts of these audit systems and how they could be improved in Dar es Salaam and Tanzania in general.

## Methods

### Study setting

The study was conducted in Dar es Salaam, a city with more than 2.5 million people, 19 hospitals, 10 health centers and 60 dispensaries (government and non-government owned) that provided maternity services [3]. The study included the four main public hospitals namely Muhimbili National Hospital, Amana, Mwananyamala and Temeke municipal hospitals, and four major private hospitals namely Aga Khan, Hindu Mandal, Mikocheni and Massana. The sample of the hospitals exceeded 30% of the health facilities recommended by WHO/UNICEF to represent a certain geographical area when assessing obstetric care services [14]. In Tanzania, the national guideline for composition of maternal mortality audit committee at the hospital level requires involvement of medical officer in-charge of the facility, head of the department of obstetrics and gynaecology, matron, obstetricians, nurse in-charge of the labour ward, pharmacist, head of the laboratory, district medical officer, district nurse officer, district reproductive and child health coordinator and all doctors present on the day of audit. In addition to this list paediatricians and nurse in-charge of neonatal unit are recommended for the perinatal mortality audit committee.

### Data collection

The study used both qualitative and quantitative methods. The qualitative component involved in-depth interviews of 29 respondents with a range of 1 - 5 purposely selected from each hospital. These were either members of maternal and perinatal audit committees or only administrators (heads of the department of obstetrics and gynaecology or in-charge of maternity wards) in places where such committees did not exist. The interviews investigated the existence, structure (composition), process and outcome of such audits in clinical practice and how they could be improved in their hospitals. The interview guide (Additional file 1) used the components of an ideal model audit to assess composition, timing and frequency of audit meetings, selection of cases, feedback provision, dissemination of recommendations, record keeping, analysis of results and use of audit recommendations for institutional planning and budgeting. The interviews were tape recorded on interviewee's consent. Immediately after the interview, the interviewers listened to the tape to clarify certain issues and confirmed that all the main points were included in the notes.

The quantitative component used a semi-structured questionnaire (Additional file 1) to interview 30 health care providers in the maternity wards, available on the day of study, to assess their awareness, attitude and practice towards maternal and perinatal death audits. In both interview categories (qualitative and quantitative) the study explored the level of implementation of audit recommendations and interviewers' suggestions for improvement of the audit systems in their respective facilities. The research team also reviewed records of the institutional deliveries, maternal and perinatal deaths, their causes as well as audited cases for 2007. The choice for 2007 records was based on the assumption that the data for 2008 had not been compiled in some of the facilities at the time of the study which started in January 2009.

The avoidable factors for audited cases were categorized according to the "3 Delays Model", defined as; phase 1: delay in decision to seek care, phase 2: delay in reaching care, and phase 3: delay in receiving care or substandard care [15]. The audit committees determined the phase of delay by establishing the time spent at home before a decision was made to seek health care after onset of complication, time spent after having made this decision to reaching care, time interval between admission and administration of treatment. This time was then compared with 1) the estimated time interval from onset of complication to death for various obstetric complications and 2) the requirements for birth preparedness and complication readiness. It is estimated that, if untreated, maternal death occurs on average in 2 hours from postpartum haemorrhage, 12 hours from antepartum haemorrhage, 2 days from obstructed labour and 6 days from infection [16]. The concept of birth preparedness and complication readiness, a strategy to promote planning for normal birth and timely use of skilled maternal and neonatal care in case of an emergency, requires a woman to seek care immediately after onset of danger signs of complications, improved accessibility of health care and prompt provision of appropriate care upon admission [17].

The audit committees reviewed the management offered to the deceased mother and determined the quality of care by comparing it with that recommended in the national management guidelines for obstetric emergencies, considered as standard care in the country. *'Substandard care' *was defined as any care considered being below acceptable standards [18]. The death was only attributed to substandard care if the management given was judged by the audit team to have contributed significantly to the death of the mother and that the standard treatment may have altered the outcome.

### Data analysis

The audio-taped interviews were transcribed and then translated from Swahili to English. A qualitative content analysis method as described by Graneheim and Lundman [19] was used to analyse the data. Analysis included thorough reading of the transcribed text to identify meaning units i.e. statements that were related to the topic of analysis. The meaning units were condensed, abstracted, coded and then categorized according to similarities and differences in content. The quantitative data from health care providers' interviews and review of institutional records were entered into the EPInfo6 program. Data was summarized by frequency tables.

### Ethical Considerations

Ethical clearance was obtained from Muhimbili University of Health and Allied Sciences, Senate Research and Publication Committee. Permission to conduct the study was obtained from the respective municipal and hospital authorities. Anonymity and confidentiality were discussed and informed verbal consent was obtained from each respondent. The tape records and written information were kept confidential and restricted to the research team only.

## Results

### Institutional Pregnancy Outcome in 2007

Of all deliveries (70,661) in these institutions 97% took place in the four government owned hospitals with the highest rate at Amana (24,862). The combined hospital-based MMR was 218/100,000 live births ranging from zero at Hindu Mandal to as high as 385/100,000 live births at Muhimbili. Of all maternal deaths which occurred in these institutions 78% were attributed to eclampsia, obstetric haemorrhage, severe anaemia, sepsis and ruptured uterus. There were 3,134 perinatal deaths, of these 57% were stillbirths and 40% were attributed to birth asphyxia, prematurity and neonatal sepsis. The causes of stillbirths were not established. The average hospital based PNMR was 44 deaths per 1000 births and ranged from 17 at Amana to as high as 147 deaths per 1000 births at the Muhimbili, the only public hospital in Dar es Salaam which had a neonatal intensive care unit during the study period (Table 1).

**Table 1:** Causes of maternal and perinatal deaths in Dar es Salaam hospitals in 2007

Parameters	MNH	Amana	M/mala	Temeke	Aga Khan	Miko- cheni	Hindu Mandal	Massana	Total
*Causes of Maternal Deaths*									
Eclampsia	12	0	3	26	1	0	0	1	43
Obstetric haemorrhage	7	5	6	11	1	0	0	0	30
Ruptured uterus	4	2	2	4	0	0	0	1	13
Severe anaemia	5	0	7	9	0	0	0	0	21
Puerperal sepsis	1	2	5	2	0	0	0	0	10
Others	7^§^	3	6	16	0	0	0	0	33
Total deaths	36	12	29	68	2	1	0	2	150
*Causes of Perinatal Deaths*									
Stillbirths	397	399	436	482	26	5	7	33	1,785
Birth asphyxia	581	5	43	46	0	1	0	0	676
Prematurity	339	6	41	9	1	0	0	0	396
Sepsis	70	5	76	14	1	0	0	0	166
Congenital malformations	1	0	2	0	4	0	0	0	7
Others	41	7	32	9	7	1	0	7	104
Total deaths	1,429	422	630	560	39	7	7	40	3,134
Total deliveries	9,743	24,862	15,464	18,490	1,094	288	316	404	70,661
MMR	385	49	193	378	187	353	0	269	218
PNMR	147	17	41	30	36	24	22	99	44

***NOTE***:

§ = of these 3 were due to ruptured ectopic pregnancy; MMR = maternal mortality ratio (deaths per 100,000 live births); PNMR = perinatal mortality rate (deaths per 1000 births); MNH = Muhimbili National Hospital; M/mala = Mwananyamala.

### Existence of maternal and perinatal death audits

Maternal death audit committees existed only in 50% (4) of the studied hospitals (i.e. Temeke, Mwananyamala, Amana and Aga Khan). At Muhimbili maternal and perinatal audit committees were established around 2002 and lasted for about 2 years. During the study period, selected maternal deaths were discussed weekly at departmental level mainly by the doctors, medical students and a few midwives. The discussion lacked documentation and disseminations of key decision points. Three respondents attributed the death of previous audit committees to the failure of the hospital administration to implement audit recommendations. This led to demoralization when the audit team repeatedly noted the same avoidable factors being associated with consecutive deaths despite strategic interventions having been recommended. In addition, one of the key respondents reported that *"there is also a serious decline of accountability and commitment among the staff, and this can be tracked from the hospital topmost administrators down to the care providers"*.

Perinatal audit committees only existed at Aga Khan, Amana and Hindu Mandal hospitals. However, the committees met occasionally on a monthly basis at Amana and two times a year at Hindu Mandal. When asked why there was no any audit committee at Mikocheni hospital, one respondent said, *"it is because most deaths had clearly known causes, for instance, intrauterine fetal deaths, fetal distress and so on"*. None of the key respondents linked the absence of such audit committees to the lack of resources like finance, supplies or human in any of the studied institutions.

### Structure of the audit committees

Municipal health managers were only involved in these committees at Temeke and Amana hospitals. However, none of the municipal medical officers, representatives from theatre or pharmacy was involved in any of the hospital audit committees. Laboratory representatives were only involved at Aga Khan hospital.

### Auditing process

Audit meetings were conducted within the first 24 hours after occurrence of death only at Aga Khan hospital. There were neither records of the key decision points nor action plan in any of the facilities where such audit systems existed to help members of the committee to track the implementation of recommendations given in the previous meetings. The audit reports were never analyzed later in any of these hospitals. All facilities with audit systems gave feedback to the responsible care providers. Audit reports in private hospitals were never disseminated anywhere beyond their hospitals. It was reported that audit recommendations were used for hospital planning and budgeting for maternal care improvement in all facilities where the system existed.

### Audit results

Of the audited maternal deaths in 2007 at Amana, Mwananyamala, Temeke and Aga Khan hospitals 69% (43) were associated with substandard care at the facility (Table 2). Almost one third (28%) of all perinatal deaths which occurred at Aga Khan hospital were audited. Of these 55% (6) were judged to be associated with phase three delay, 18% (2) phase two delay and 27% (3) phase one delay.

**Table 2:** Phases of delay for mothers died in Dar es Salaam hospitals in 2007

Phase of delay for audited maternal deaths	Aga Khan n = 2	Amana n = 12	M/mala n = 29	Temeke n = 68	Total n = 111
Phase 1	0	0	4	11	15
Phase 2	0	3	1	0	4
Phase 3	2	6	13	22	43
					
Total audited deaths (%)	2 (100%)	9 (75%)	18 (62%)	33 (49%)	62 (56%)

**Note**:

n = total number of maternal deaths which occurred in the specified health facility. Phase 1: delay in decision to seek care, phase 2: delay in reaching care and phase 3: delays in receiving care or substandard care

### Awareness, knowledge, attitude and practice towards maternal and perinatal death audits

The level of awareness of maternal and perinatal death audit committees was as high as 87% (26) among the interviewed health care providers. On the contrary, half of the respondents from Amana hospital were not aware of the existence of such committees in their institution. Of the respondents from the five facilities with maternal and/or perinatal death audit systems more than one third (35%) reported that the objectives of such audits had not been communicated to all care providers in the department, and the figure was as high as 50% at Amana, Mwananyamala and Temeke hospitals.

Only 60% (12) of all respondents from the institutions where maternal and/or perinatal death audits existed, were aware of at least one recommendation which had been provided by either maternal or perinatal death audit committee in their hospitals. Similarly, only 40% (8) of the respondents in these facilities remembered and mentioned at least one action that was implemented in the hospital because of maternal or perinatal audit committee recommendations. The few mentioned actions taken as a result of the audit recommendations at Temeke and Amana included writing of statements, internal transfer and removal from supervisory roles of the staff that were held responsible for the deaths. All 30 (100%) respondents, including those from places where maternal and perinatal death audits did not exist, believed that audits can affect how people conduct maternal and newborn care anywhere including their hospitals (Additional file 2).

## Discussion

### Factors for absence of audit committees

Maternal and perinatal audit systems have emerged as a fundamental principle in the context of obstetric care in the world over the recent years. Clinical audit systems improve patient care and service delivery, enlighten care providers on their strengths and weaknesses, improve knowledge and behaviour change in their patient care and enhance cost-effective use of resources [9,20,21]. The absence of maternal and perinatal audits in 50% and 63% of the hospitals respectively could be mainly linked to the lack of commitment, dedication and accountability of key staff and leadership. The reasons given in some facilities for the absence of such an important tool indicate that the philosophy for this tool has not been well conceptualized. The absence of such audit systems in the teaching hospitals like Muhimbili, Mikocheni and Massana, suggests lack of emphasis on clinical audits in their curricula, lack of evidence-based training and that their graduates are less likely to take lead for the same at their future working places. On the contrary reports from other African countries show that such audits are largely found in larger hospitals and academic institutions [22].

Such unacceptably slow pace of replicating proven best practices in this region is worrisome and suggests poor leadership performance. Leadership is about change, and is all about getting things done [23]. Carrying out "*business as usual*", lack of proactivity and a static mindset among the key actors and poor supervision of health systems are progress blocking agents which have been reported as the leading factors for poor performance of health sectors in sub-Saharan Africa [24]. These findings call for a more proactive and dedicated leadership at national and institutional levels.

### A need for effective audit systems in Dar es Salaam institutions

The fact that 69% of maternal and 55% of perinatal deaths were related to substandard care at the facility level indicates a high degree of poor obstetric care and a need for effective audit systems. Similar findings have been reported from within health institutions in other low income countries indicating that 50% - 77% of all perinatal deaths are avoidable through treatment of common conditions, closer monitoring and skillful management of labour [25-27]. The findings that the leading causes of maternal death were eclampsia, haemorrhage and severe anaemia indicate conditions that can be prevented by improving the quality of care through early detection of danger signs and prompt treatment.

### Audit committees' structural factors

Failure to take part of the key decision makers like the municipal and hospital medical officer in charge and other administrators in some of the municipal hospitals' audit committees (as recommended by the Ministry of Health of Tanzania) raised questions about how well the decisions made during deaths' review meetings were adopted into municipal and hospital plans. Although this category of audit members may not be technical during audit discussions, they have been reported to have a big role to play when it comes to implementation of the key points made for change. Lack of key hospital decision makers in the audit committees at Muhimbili which were established in the early 2000 s was linked to poor implementation of audit recommendations, disappointment and ultimately death of the committees. Quite often the same administration-related factors were linked to maternal deaths and various recommendations had been discussed over and over yet without implementation. In places where establishment of maternal and perinatal audits have led to improved quality of obstetric care, the success has been particularly attributed to the process of accountability of both health providers and key decision makers [9,28]. Clinical audit must be well structured, conducted according to acceptable principles and there must be commitment to the process from a care provider through the health managers and policy makers, otherwise it is unlikely to bring change [8,20,29].

### Change factors in the audit process

The absence of records of the key decision points, recommendations and action plans, as well as lack of regular analysis of the audit reports in any of the facilities where audits were reported to exist, indicates poor documentation and poor information management systems. Commonly, action plans help members of the committee to track the implementation of recommendations given in the previous meetings. Usually, quarterly to six-monthly analysis of audit systems is recommended for evaluation of recurrence of substandard care factors and the success of implementation of the recommendations [30]. The lack of documentation at maternal death discussions at the national hospital and failure of the private hospitals to disseminate their audit reports to the city and national authorities indicate lack of links with the overall policy making authorities. This denies these organs from important information to base their decisions and policy making upon.

### Potential factors for efficient audit systems

The high level of awareness, knowledge and positive attitudes (83 - 100%) towards maternal and perinatal audit committees among the care providers including those from places where such committees did not exist, indicate substantial acceptability, readiness and willingness for change in service provision in these institutions. Failure of care providers (40 - 60%) to mention at least one recommendation or any action which had ever been taken following audit suggests either lack of recommendations formulation during audit or poor implementation. The fact that up to 82% of the recommendations made during audits in other African countries are implemented [31] indicates a need to improve efficiency of the existing audit systems in Dar es Salaam hospitals. The reported punishment of staff held responsible for the deaths following audit may lead to incorrect information in future incidences and create conflict among staff and should be strongly discouraged [9].

Considering these factors and the fact that Tanzania is already off-track for the maternal mortality-related Millennium Development Goal with MMR of 529/100,000 live births in 1995 and 578/100,000 live births in 2005 [32] evidence-based John Kotter's eight-step process for implementing successful changes in any organization is indicated for effective audit systems in this region. These steps are: to create a sense of urgency for change, create powerful group guiding the change, develop and communicate the change vision and strategy, empower others to act, produce short-term wins, press harder and faster after the first successes and create a new culture for sustainability [33].

## Conclusions

Clinical audits are greatly rewarding for patients and health providers. These are within reach in low income countries like Tanzania, but they are just not being done or conducted ineffectively in most institutions. The existing maternal and perinatal audit systems in Dar es Salaam health institutions are still poorly established in structure and process; and are less effective to improve the quality of care. Fundamental changes are urgently needed for successful audit systems in these institutions.

## Abbreviations

H/Mandal: Hindu Mandal hospital; M/mala: Mwananyamala hospital; MMR: Maternal mortality ratio; MNH: Muhimbili National Hospital; PNMR: Perinatal mortality rate; UNICEF: The United Nations Children's Fund; WHO: World Health Organization.

## Competing interests

The authors declare that they have no competing interests.

## Authors' contributions

ASN participated in design of the study, data collection, analysis and drafted the manuscript. DPU participated in design of the study, data collection and the analyses and reviewed the manuscript. ABP participated in design of the study and reviewed the manuscript. FK participated in data collection and reviewed the manuscript. JVR participated in design of the study and reviewed the manuscript. All authors read and approved the final manuscript.

## Pre-publication history

The pre-publication history for this paper can be accessed here:

http://www.biomedcentral.com/1471-2393/10/29/prepub

## Supplementary Material

Additional file 1 **Instruments I and II for data collection**. Instrument I: Topic guide for the in-depth interview of the members of the maternal and perinatal death audit committees. Instrument II: a questionnaire for interview of health workers working in maternity wards on maternal and perinatal audit systems Click here for file

Additional file 2 **Table 3: Awareness of, knowledge, attitude and practice towards maternal and perinatal death audits among the health care providers in maternity wards**. Awareness of, knowledge, attitude and practice towards maternal and perinatal death audits among the health care providers in the maternity wards at Muhimbili National Hospital, Amana, Hindu Mandal, Mwananyamala, Mikocheni, Temeke, Massana and Aga Khan hospitals. Click here for file
