# Supplementary material for: Factors for change in maternal and perinatal audit systems in Dar es Salaam hospitals, Tanzania
Source: BMC Pregnancy Childbirth. 2010 Jun 3;10:29. doi: 10.1186/1471-2393-10-29 (PMC2896922; doi:10.1186/1471-2393-10-29)
Supplement: Additional file 1 — Instruments I and II for data collection. Instrument I: Topic guide for the in-depth interview of the members of the maternal and perinatal death audit committees. Instrument II: a questionnaire for interview of health workers working in maternity wards on maternal and perinatal audit systems [file 1471-2393-10-29-S1.DOCX]

# INSTRUMENTS FOR DATA COLLECTION

# Instrument I

# TOPIC GUIDE FOR THE IN-DEPTH INTERVIEW OF THE MEMBERS OF THE MATERNAL AND PERINATAL DEATH AUDIT COMMITTEES

Start after self introduction and getting the consent from the respondent.

1. Experience of conducting maternal/perinatal mortality audit

*(Probe for more information on responsibilities, involvement in the maternal/ perinatal mortality audit committees in this hospital, for how long s/he has been involved in the committee)*

1. How is the audit process conducted?

*(Probe for information on (who initiate the process of auditing?, how soon after the death is an audit conducted? how frequently are the audits conducted, who are involved in the audit process (their professionalism and managerial positions), Do you conduct audit on all deaths or only some? If only some. how are the deaths selected for discussion ,what materials are used for documenting the process e.g. case notes, antenatal cards, partograms, delivery records etc? availability of special forms, review of the case, Who are the participants in a typical case of auditing? how do they reach consensus on recommendation, How long does an audit take to complete? Who analyses the results of an audit?*  *How feedback to the care provider is given, and record keeping)*

1. How are the analyzed results and recommendations of the audit committees connected to the institutional database? *(Interviewer: Probe for information about how the information on maternal and perinatal audits are included in the institutional database)*
2. How are recommendations of the audit committee disseminated to the stakeholders?

*(Probe for information if the audit information is also disseminated to the peer health care providers within the hospital and referring one, institutional administration, Ministry of Health, the City medical officer for health as well as the community).*

1. How are the audit recommendations used by the hospital managers and health policy makers in planning and budgeting in order to implement changes in the hospital?

*(Probe for more information about the use of the recommendations in planning, budgeting in order to implement changes in the hospital, examples of the changes that have ever been implemented as a result of recommendation of the audit committee since its establishment, reasons for failures).*

1. What are your opinions on how audit is conducted and its impact on improving care?

*(Probe on how the audit process can be improved to bring change in improving care)*

1. Do you have any other suggestions to improve the audit mechanism in order to bring changes in obstetric care in the hospital?

**Instrument II**

**A QUESTINNAIRE FOR INTERVIEW OF HEALTH WORKERS WORKING AT MATERNITY WARDS ON MATERNAL AND PERINATAL AUDIT SYSTEMS**

***Instructions:*** *Circle the appropriate code or fill the responses of the interviewee in the spaces*

*provided after the question.*

| **Part I: Identification** | |
| --- | --- |
| 1 | Name of the Hospital ________________________________________________________ |
| 2 | Health worker’s category 01 Doctor 02 Nurse 03 Other (specify)  ________________________________________________ |
| 3 | Section of the hospital 01 Maternity 02 Neonatal unit 03 Administration |
| **Part II: Awareness** | |
| 4 | Are you aware of maternal and perinatal audit committee? 01 YES 02 NO |
| 5 | If Yes, please mention the reasons for establishment of such committees?  i. ____________________________________________________________________________  ___________________________________________________________________________  ii. ____________________________________________________________________________  ____________________________________________________________________________ |
| 6 | Do you have such committees in this hospital? 01 YES 02 NO 03 DON’T KNOW |
| 7 | What type of committee among the two do you have?  01 Maternal audit 02 Perinatal audit 03 Both |
| 8 | Do you remember how such committees were introduced in this hospital? 01 YES 02 NO |
| 9 | If yes in qn 7, Explain  _____________________________________________________________________________ |
| 10 | Do you know the main objective or vision of these committees? 01 YES 02 NO |
| 11 | If yes what is it? ________________________________________________________________ |
| 12 | Are there core/constant members of these committees that you know? 01 YES 02 NO |
| 13 | If yes, mention few  1. ______________________________ 3. ___________________________  2_______________________________ 4. ___________________________ |
| 14 | Have the objectives of these committees been communicated to all members of the staff working in maternity wards? 01 YES 02 NO 03 DON’T KNOW |
| **Part III: Attitude** | |
| 15 | Do you think audit committees can affect how people conduct maternal and newborn care anywhere? 01 YES 02 NO |
| 16 | Do you think audit committee can affect how you conduct maternal and newborn care in this hospital? 01 YES 02 NO |
| **Part IV: Practices** | |
| 17 | Do you know any recommendation that has been provided by maternal/perinatal death audit committee in this hospital? 01 YES 02 NO |
| 18 | If Yes, Mention them  1. _______________________________________________________________________  2. _______________________________________________________________________ |
| 19 | Do you remember any action that was taken in this hospital because of maternal/perinatal audit committee recommendation? 01 YES 02 NO |
| 20 | If Yes, Mention them  1. _______________________________________________________________________  2. _______________________________________________________________________  3. _______________________________________________________________________ |
| 21 | Have you ever seen any effect on how maternal and newborn care is been provided in this hospital because of audit committee recommendations? 01 YES 02 NO |
| 22 | How could functions of these committees be improved? Explain |
