# Supplementary material for: Factors for change in maternal and perinatal audit systems in Dar es Salaam hospitals, Tanzania
Source: BMC Pregnancy Childbirth. 2010 Jun 3;10:29. doi: 10.1186/1471-2393-10-29 (PMC2896922; doi:10.1186/1471-2393-10-29)
Supplement: Additional file 2 — Table 3: Awareness of, knowledge, attitude and practice towards maternal and perinatal death audits among the health care providers in maternity wards. Awareness of, knowledge, attitude and practice towards maternal and perinatal death audits among the health care providers in the maternity wards at Muhimbili National Hospital, Amana, Hindu Mandal, Mwananyamala, Mikocheni, Temeke, Massana and Aga Khan hospitals. [file 1471-2393-10-29-S2.DOCX]

**Table 3: Awareness of, knowledge, attitude and practice towards maternal and perinatal death audits among the health care providers in the maternity wards.**

| **Variables** | MNH  n = 4 | Amana  n= 4 | Hindu  Mandal  n= 4 | M/mala  n= 4 | Mikocheni  n=3 | Temeke  n= 6 | Massana  n= 3 | Aga  Khan  n= 2 | Total  n = 30 |
| --- | --- | --- | --- | --- | --- | --- | --- | --- | --- |
| ***Awareness and knowledge*** |  |  |  |  |  |  |  |  |  |
| Aware of maternal and perinatal death audits | 3 | 2 | 4 | 4 | 3 | 6 | 2 | 2 | 26 |
| Mentioned at least one reason for establishing such audits | 2 | 2 | 4 | 4 | 3 | 6 | 2 | 2 | 25 |
| Aware of the presence of audit committee in their facility | NA | 2 | 4 | 4 | NA | 6 | NA | 2 | 18 |
| Knew the main objective or vision of the audits | NA | 2 | 4 | 2 | NA | 6 | NA | 2 | 16 |
| Reported that objectives of their audit had been communicated to all care providers in maternity wards | NA | 2 | 4 | 2 | NA | 3 | NA | 2 | 13 |
| ***Attitudes*** |  |  |  |  |  |  |  |  |  |
| Believed that audit can affect how people conduct maternal and newborn care anywhere | 4 | 4 | 4 | 4 | 3 | 6 | 3 | 2 | 30 |
| ***Practices*** |  |  |  |  |  |  |  |  |  |
| Knew at least one recommendation that had been provided by maternal/ perinatal death audit committee in this hospital. | NA | 1 | 2 | 3 | NA | 5 | NA | 1 | 12 |
| Remembered at least one action that was implemented in the hospital because of maternal/perinatal audit committee recommendations. | NA | 1 | 0 | 1 | NA | 6 | NA | 0 | 8 |
| Mentioned at least one action that was implemented in her hospital because of maternal/perinatal audit committee recommendation. | NA | 1 | 0 | 1 | NA | 6 | NA | 0 | 8 |
| Ever seen any effect on maternal and newborn care in the hospital because of audit committee recommendations | NA | 1 | 2 | 2 | NA | 5 | NA | 1 | 11 |

**NOTE:** *MNH = Muhimbili National Hospital, M/mala = Mwananyamala, NA = not applicable (there was neither maternal nor perinatal death audit committee. This also included MNH which discussed the deaths at departmental level but was not considered as an audit committee).*
